# Advancing thermal management in electronics: a review of innovative heat sink designs and optimization techniques

**DOI:** 10.1039/d4ra05845c

**Published:** 2024-10-16

**Authors:** Md Atiqur Rahman, S. M. Mozammil Hasnain, Prabhu Paramasivam, Abinet Gosaye Ayanie

**Affiliations:** a Department of Mechanical Engineering, Vignan's Foundation for Science, Technology & Research (Deemed to Be University) Vadlamudi Guntur Andhra Pradesh 522213 India rahman.md4u@gmail.com; b Marwadi University Research Center, Department of Mechanical Engineering, Faculty of Engineering & Technology, Marwadi University Rajkot 360003 Gujarat India smmh429@gmail.com prabhu.paramasivam@marwadieducation.edu.in; c Department of Mechanical Engineering, Adama Science and Technology University Adama – 2552 Ethiopia abinet.gosaye@astu.edu.et

## Abstract

The ongoing trend towards miniaturizing electronic devices and increasing their power densities has created substantial challenges in managing the heat they produce. Traditional heat sink designs often fall short of meeting modern electronics' rigorous thermal management needs. As a result, researchers and engineers are turning to innovative heat sink designs and optimization methods to improve thermal performance. This review article offers an overview of the latest advancements in designing and optimising advanced heat sinks for electronic cooling. It explores various techniques to enhance heat transfer, including advanced surface geometries and microchannels. Additionally, the review covers computational fluid dynamics (CFD) modelling and experimental validation methods used to refine heat sink designs. Further insight into fouling and its prevention has been discussed. The insights provided in this article are intended to serve as a valuable resource for thermal engineers, and researchers focused on managing the heat in high-power electronic systems.

## Abstract 2

The ongoing trend towards miniaturizing electronic devices and increasing their power densities has created substantial challenges in managing the heat they produce.

## Graphical abstract

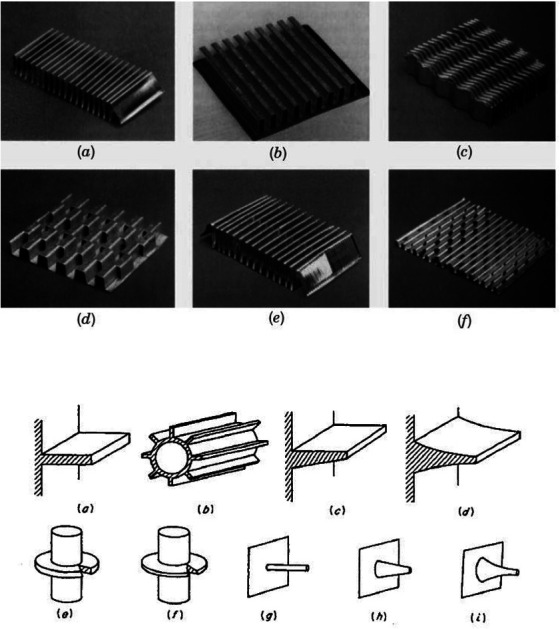

## 1. Introduction

A mechanism that facilitates the HT between physical systems by utilizing various methods of HT, such as conduction, convection, or radiation, is referred to as an HX. The HT within a HX may occur within a single solid material or between multiple solid materials, between a solid material and a fluid, or between multiple fluids.^1–4^ HXs are utilized in various fields such as household applications, aerospace, oil and gas, automotive, HVAC, wastewater treatment, cryogenics, manufacturing, and other industries. Increasing energy costs, resource constraints, material efficiency, and the need for compact designs have led to the development of improved HXs.^5^ Hence HT enhancement is crucial, especially in engineering application, as it can significantly improve the efficiency of processes, reactors, and separation units. Here are several methods and technologies used to enhance HT in engineering applications:

• Heat exchanger design.

Extended surface heat exchangers: utilizing fins or extended surfaces increases the HT area, enhancing the heat exchange rate.

Plate heat exchangers: compact and efficient, these use thin plates to transfer heat between two fluids.

Shell-and-tube heat exchangers: commonly used in chemical engineering, optimizing the tube arrangement and shell design can improve HT efficiency.

• Use of enhanced tubes and channels.

Corrugated or helical tubes: these create turbulence and improve HT by disrupting the boundary layer.

Structured packing: in distillation or absorption columns, structured packing increases surface area and enhances mass and HT.

• Nanofluids.

Nanoparticle addition: adding nanoparticles to fluids can significantly increase thermal conductivity and *h*_m_. Examples include adding metallic or metal oxide nanoparticles to base fluids.

• Turbulence promoters.

Additives: inserts like vortex generators or turbulators can be added to fluid pathways to enhance turbulence and improve HT.

Swirl flow: inducing swirl in flow paths can improve mixing and HT.

• Phase change materials (PCMs).

Heat storage: PCMs can store and release large amounts of heat during phase transitions, which can be used to regulate temperature and improve overall heat transfer efficiency.

• Heat transfer fluids.

Optimized fluids: using fluids with higher thermal conductivity or specific heat capacities can improve HT. For example, selecting the right heat transfer fluid in reactors or heat exchangers is crucial.

• Microchannel technology.

Microchannel heat exchangers: these use tiny channels with hydraulic diameter ≤ 1 to enhance heat transfer through higher surface area-to-volume ratios and improved HT rates due to laminar flow conditions.

• Surface modifications.

Textured surfaces: coating or texturing surfaces (*e.g.*, using roughened or patterned surfaces) can enhance HT by increasing turbulence and reducing thermal resistance.

• Improved mixing techniques.

Static mixers: adding static mixers to process streams can improve mixing and thus enhance HT.

Agitation: in reactors, agitation or stirring can enhance HT by improving mixing.

• Heat transfer enhancement in reactors.

Internal fittings: in chemical reactors, adding fittings or baffles can enhance HT by disrupting flow patterns and promoting better mixing.

Catalyst design: optimizing catalyst placement and distribution in catalytic reactors can improve heat transfer and reaction rates.

• Energy integration and recovery.

Heat integration: employing heat integration techniques like heat cascades or heat recovery networks to utilize excess heat from one process in another can enhance overall energy efficiency.

Microchip technology has enabled an expanding amount of electronic components to be placed on one chip. The shrinking of microchips, coupled with a rise in the number of electronic parts per chip, has heightened the levels of heat that the microchips endure. This has created a necessity for improved heat sinks that can effectively remove the high heat levels from the electronic components to ensure their safety and reliability. Furthermore, progress in developing newer generations of microprocessors, batteries, converters, and various other small-scale devices has been slowed down by the constraints of existing cooling technology. These limitations serve as a roadblock to advances in computing power, energy storage, and other capabilities. Industries like electric vehicles (EVs), hybrid electric vehicles (HEVs), and plug-in hybrid electric vehicles (PHEVs) are exploring innovative solutions in small-scale cooling techniques to break through these barriers and create cooling systems that are both effective and compact.^6^

Advancements in HT could involve a surge in the heat load for the same HX area, a decrease in dimensions pumping energy needed for the identical thermal load, or a reduction in the temperature difference during operation for the identical thermal load. The temperature change is a crucial factor in transferring heat. One option to improve the *h*_m_ on the gas side is to increase airflow. However, this requires a more powerful pump, increasing energy consumption and costs. One way to augment the HT capacity on the gas side of an HX is to expand the surface area. This can be achieved by adding extra/additional surfaces, such as fins, to the primary surface.^7,8^

**Fig. 1:**
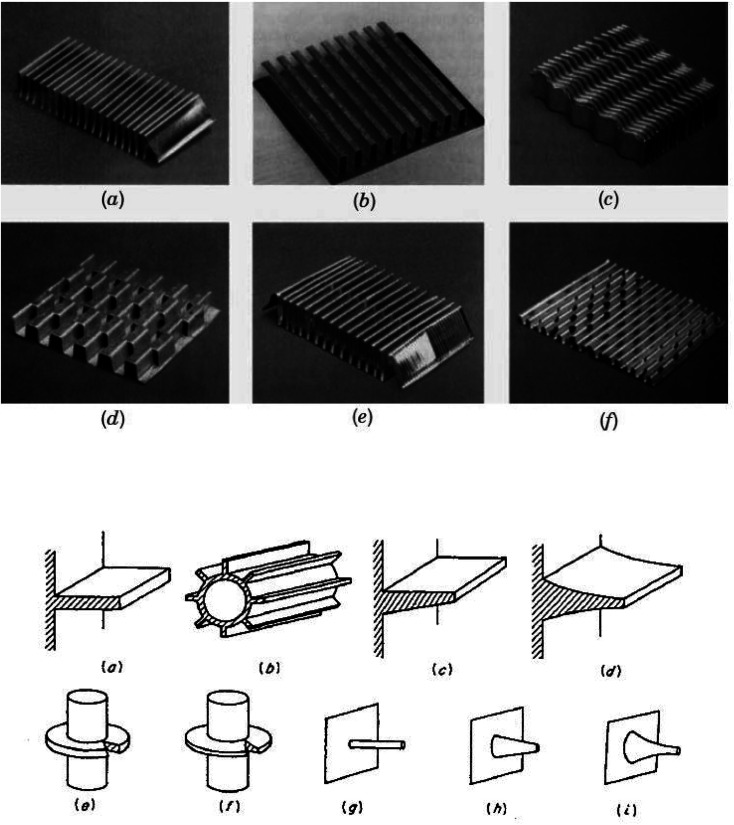
(a) Different fin shapes. Reproduced with permission^9^ copyright © 2003, John Wiley and Sons. (b) Extended surfaces. Reproduced with permission^11^ copyright © 2001, John Wiley and Sons.

Adding extensions to a foundation area can typically enhance its HT region by seven to fifteen times.^9^ HT occurs from the base layer to the extensions through conduction and from the extensions to the surrounding air through convection. Extensions are created from highly heat-conductive materials like aluminum and copper, while brass or stainless steel extensions are used for harsh or high-temperature environments.^10^ The material's thermal conductivity must be high to minimize temperature differences from the base to the tip of the extension. Additionally, the extensions' thickness should be smaller than their length to reduce temperature variations. Thinner extensions are preferred to maintain a light weight per unit length of the HX, although a minimal thickness is necessary for structural integrity. The foundation can have a flat or cylindrical shape, while extensions can come in various shapes, like longitudinal profiles (trapezoidal, rectangular, or parabolic) or pin fin profile (cylindrical, cone or spline). Extensions (adhesives, welding, extrusion, soldering, or brazing) can be attached to the base layer. Examples of typical extension profiles are illustrated in Fig. 1a.

It should be noted that fins may not solely serve to enlarge the HT area; they may also impact the HT rate. Types of such fins include curved strips, angled, undulating, and punctured, as demonstrated in Fig. 1b. The augmentation in the HT rate with these iterations of a smooth fin results from modifications in the flow pattern. It can be linked to the disturbance of the BL, swirl (rotational flow) flow and blending, and decrease in thermal resistance due to reduction in the thermal BL.

The failure rate of electronic parts changes significantly with temperature, and generating too much heat can lead to high temperatures that can harm the electronic part's safety, dependability, and functionality.^12^ Removing excess heat from electronic parts to ensure their trustworthiness and protection is crucial, as reducing the integrated circuit (IC) junction temperature by 10 °C can cut the failure rate in half.^13^

Heat sinks are used to eliminate excess heat and regulate the temperature of an electronic device within safe limits. Various factors such as the amount of excess heat, device safety, space constraints, cost, reliability, usability, manufacturability, compatibility, and performance determine the type of heat sink used for an electronic device.^13,14^

• Convection (natural) with/without radiation in rectangular enclosures, arrays of pin fins or plates, and vertical/inclined parallel plate channels.

• Convection (forced) with liquid/air as a coolant in a rectangular duct having a heating source mounted (flush) or protruding and fin arrays of plate or pins.

• Convection (forced) with mixed heat sinks and jet impingement heat removal.

• Cooling using PCM, immersion, pool boiling, heat pipes, evaporation, boiling forced convection.

• Microchannel with porous media, two/single phase cooling.

• Cooling systems powered by refrigerant heat pumps, passive cooling, heat dispersion through thermoelectric principles, and innovative cooling agents like nanofluids use dielectric or non-dielectric liquids.

A heat sink that utilizes forced air convection as a cooling method is suitable for removing moderate amounts of heat in electronic devices. This is because of the accessibility of air, comfort of production, manoeuvre, maintenance, durability, affordability, safety, dependability, and uncomplicated design. However, the effectiveness of HX through fins is limited by fin efficiency, so new fin profiles are now investigated to upsurge the HT area and enhance the *h*_m_.

Three distinct approaches to enhancing heat transfer are passive, active, and composite. The active technique relies on external power, while the passive approach does not, and the composite method combines both. All three methods aim to induce turbulent flow, enhancing molecules' interaction at different temperatures and improving heat transfer efficiency.^15^ Numerous active techniques have been reported, such as the impact of an electrostatic field,^16^ swirling impinging jets,^17^ transverse mechanical oscillations,^18,19^ and magnetic field-induced swirling flow.^20^ Because of problems such as expenses, noise, security, and dependability linked with active techniques, they have not gained as much attention in the commercial sector as passive techniques.^21^ Passive techniques involve dispersing nanoparticles^22–26^ or modifying/altering HT surface properties such as adding coatings or creating rough textures, using extended surfaces to disrupt the flow and generate turbulence by incorporating inserts or secondary flow mechanisms (using swirled inserts),^27–41^ or employing coiled tubes.

The study explores methods to improve heat transfer in electronic cooling heat sinks by incorporating secondary flow mechanisms like wing/winglet vortex generators and modifying surface roughness with concavities and convexities.

Secondary flow augmentation techniques intentionally introduce additional local flow patterns within the heat exchanger alongside the primary flow. It can be challenging to differentiate between the primary and secondary flow as they often interact. One standard method of creating secondary flow is altering the HT surface geometry with VG, eliminating the need for external power input. The secondary flow facilitates mixing fluids (hot and cold), leading to a thinner thermal BL at the wall. This leads to lower thermal resistance and enhances local *h*_m_.^42,43^ In the upcoming sections, this study examines two kinds of secondary flow initiators: winglet and surface protrusion (concavity/convexity).

## 2. Optimization of heat sink shape

**Fig. 2:**
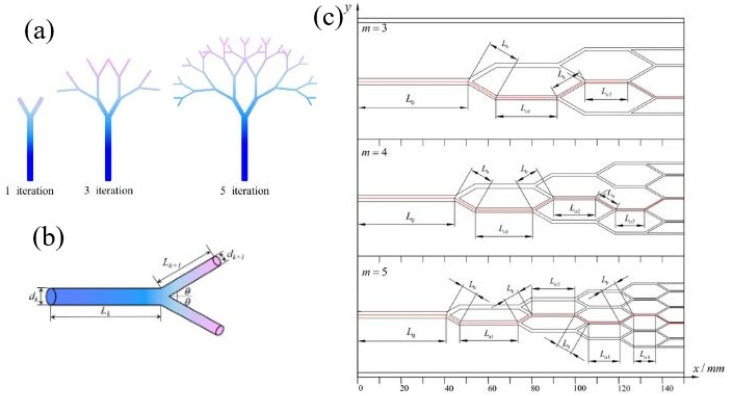
Sketch of (a) tree-like bifurcated networks and (b) the *k*th bifurcating level with *n* = 2. (c) Two-dimensional view of the tree-like bifurcating microchannels with *m* = 3, 4, 5. Reproduced with permission^46^ copyright © 2023, MDPI.

As miniaturization and the integration of microelectronic devices advance, the heat flux through chips has surged dramatically, posing significant challenges for thermal management. Researchers have extensively explored the use of microchannel heat sinks, renowned for their superior heat transfer capabilities and compact size, since their introduction by Tuckerman and Pease.^44^ Despite their advantages, conventional straight microchannels have limitations in heat transfer efficiency due to a limited range of influencing factors. To address the increasing demand for effective heat flux removal, further enhancements are essential. Various strategies have been employed to optimize the topology of straight channels, such as tree-shaped,^45,46^ wavy,^47,48^ and honeycomb-shaped microchannels,^49,50^ among others as seen in Fig. 2.

**Fig. 3:**
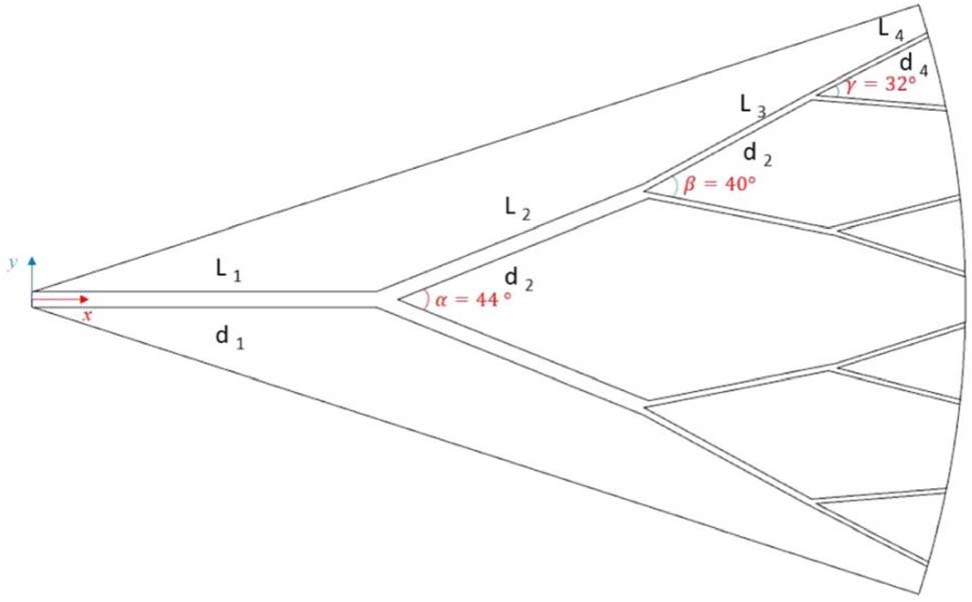
The schematic geometry of the studied fractal microchannel. Reproduced with permission^51^ copyright © 2020, Scientific Reports.

Lyu *et al.*^51^ numerically studied fluid flow and heat transfer within a fractal microchannel heatsink (Fig. 3) to promote uniform temperature distribution. The thermal performance of single-walled CNTs (SWCNT) and multi walled CNTs (MWCNT) dispersed in water and kerosene as base fluids in a fractal microchannel is investigated at Re ranging from 1500 to 3000. The findings reveal that a fractal silicon microchannel achieves uniform temperature distribution. At the highest Re number, water-based nanofluid exhibits a Nu of 20.9 and a pumping power of 0.033 W, while kerosene-based nanofluid shows a Nu of 6 and a pumping power of 0.054 W under the same conditions. The results also indicate that SWCNTs significantly enhance the Nu compared to MWCNTs, with the effect becoming more pronounced as the Re and nanoparticle vol% increase.

**Fig. 4:**
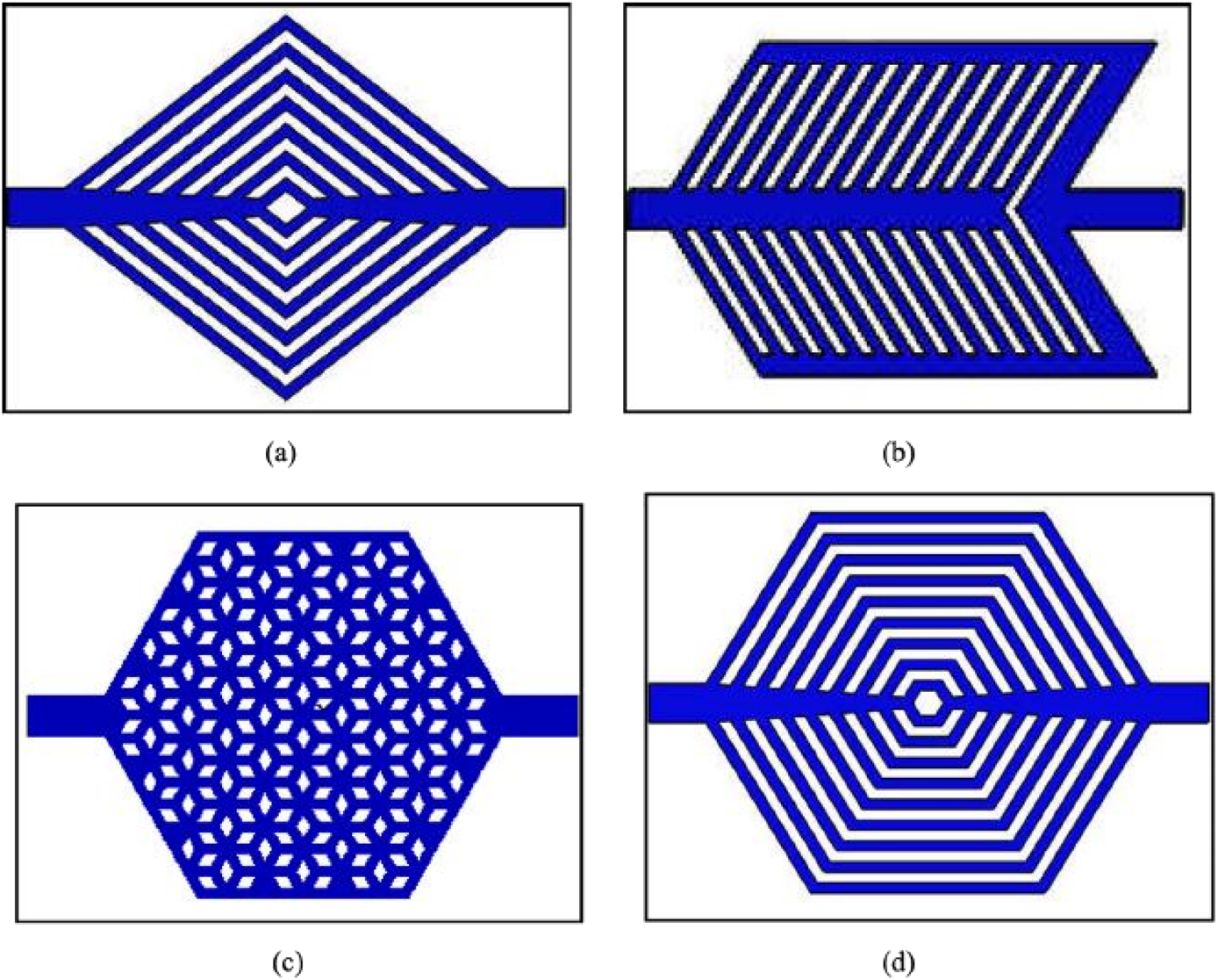
Four microchannel models (a) ternate veiny, (b) lateral veiny, (c) snowflake shaped, (d) spider netted. Reproduced with permission^52^ copyright © 2018 Elsevier.

Tan *et al.*^52^ experimentally examined how different microchannel topologies affect heat transfer performance in chip cooling systems. Initially, four distinct microchannel topologies were designed: ternate veiny, lateral veiny, snowflake-shaped, and spider-netted (Fig. 4). The experimental results demonstrated that the spider-netted microchannel outperformed the straight microchannel in heat transfer efficiency. Specifically, the maximum temperature difference at a heat flux of 100 W cm^−2^ reached 9.9 °C, with the disparity increasing further as the heat flux rose. This study concluded that the microchannel topology significantly influences heat transfer performance, particularly under high heat flux conditions.

## 3. Secondary flow initiators (VGs)

This section outlines the common traits of VG using wing/winglet. It covers various VG types, wing/winglet orientations, vortices (longitudinal/transverse), and primary/secondary vortices. Subsequently, it delves into a review of studies on wing/winglet VGs for improving HT.

### 3.1 Characteristics of VG of wing/winglet type

VGs of the wing or winglet variety have been extensively studied to augment HT by manipulating secondary flows.^53^ These devices represent a passive approach to heat transfer enhancement, in contrast to primary flow modification techniques that alter the overall flow characteristics.^54^

Typically oriented at a high *β* relative to the incoming flow, a wing or winglet VG induces flow separation at the suction side due to an opposing Δ*p*. This flow separation then gives rise to a 3D vortex flow downstream. The direction of the flow (local secondary), whether toward the impinging surface (downwash) or away from the surface (upwash), influences the local HT.

**Fig. 5:**
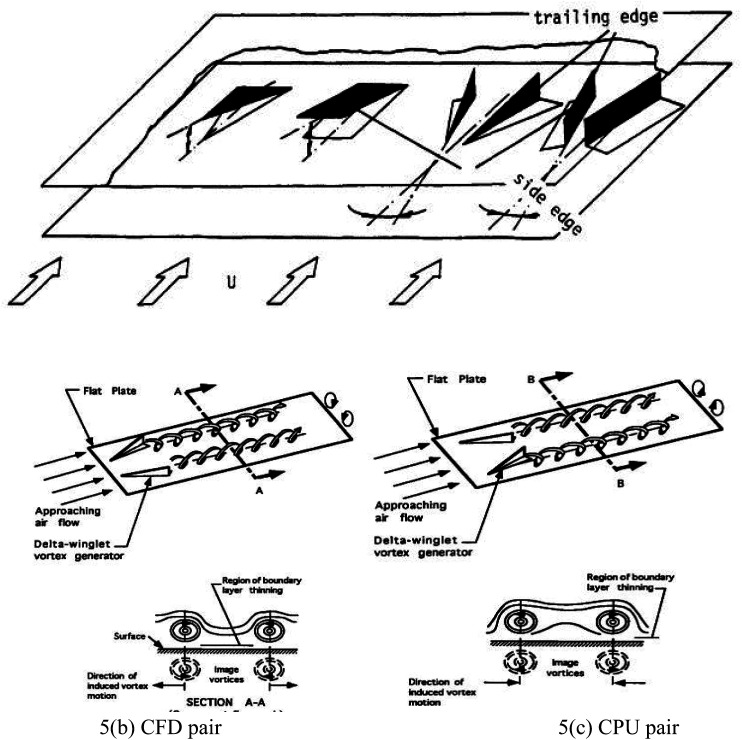
(a) Generic VGs. Reproduced with permission^55^ copyright © 1991 Elsevier. (b) CFD pair, (c) CPU pair. Reproduced with permission^54^ copyright © 1995 Elsevier.

VGs comprising rectangular wings, delta wings, rectangular winglets, and delta winglets^55^ (as depicted in Fig. 5a and b) represent a popular passive approach for enhancing HT. These devices effectively generate secondary flows, making them preferred over primary flow modification techniques.

The critical distinction between wings and winglets is their mounting orientation relative to the surface. Wings typically have their mounting edge at the trailing edge, while winglets are attached along their chord about the surface.^55^ Two winglet vortex generators can be positioned in a CFD or CFU arrangement.

In the CFD configuration, the space between the leading edges of the winglets is lesser than the distance between their trailing edges (Fig. 5a). Conversely, the leading edge spacing in CFU orientation is larger than the trailing edge spacing (Fig. 5c). The CFD arrangement creates a downwash between the counter-rotating vortices (Fig. 5b and c), while the CFU configuration results in an upwash between the vortices and a region of downwash outside the vortices in the spanwise direction.

For optimal thermal efficiency, generating the vortex close to the wall is beneficial to disrupting the laminar sublayer within a turbulent BL.^54^ Depending on the dimensions, configuration, and *β* of the VGs, the generated vortices can be categorized as either transverse or longitudinal. Longitudinal vortices with axes parallel to the main airflow direction exhibit a 3D structure. Conversely, transverse vortices with axes perpendicular to the main airflow direction remain in a 2D form.^55^

It is noteworthy that longitudinal vortices can persist over longer distances in the downstream flow direction, which can lead to enhanced heat transfer.^46,56,57^ In addition to the predominant longitudinal vortex, smaller transverse vortices may form at the leading and trailing edges, along with a horseshoe vortex at the base of the winglet. Although these secondary vortices are weaker in strength than the primary vortex, they play a significant role in the flow dynamics.^49,55^

Secondary vortices, particularly at the winglet junction where Δ*p* exist between the stagnant fluid and the boundary layer flow, contribute to the overall aerodynamic performance.^55,56^ Induced vortices near the VG trailing edge base result from flow rolling caused by low pressure in that area. Due to their proximity to the mounting surface, these secondary vortices decay quickly due to the influence of fluid viscosity.^56^

VGs can be either surface-mounted or integrally part of the surface. When punching out a VG, it is advisable to do so on the Δ*p* side of the winglet to maintain the strength and effectiveness of the resulting vortex.^57^

#### 3.1.1 VGs over a flat plate or channel flow

In inflows confined by walls, such as conduit/duct flows, a favourable Δ*p* gradient exists along the flow downstream direction, allowing a vortex within the flow to resist dissipation over longer distances.^41,58,59^ In their experimental study on channel flows, Fiebig *et al.*^55^ observed that the vortex created by the VG (wing) exhibited an elliptical cross-sectional shape. In contrast, the vortex created by the winglet persisted to be circular. Furthermore, at *β* of 40° on a delta wing, a rise in Re from 1815 to 3630 led to a deterioration of the well-defined structure of the vortex core, primarily due to significant local diffusion and low axial velocity in the core region. The researchers found that the induced drag did not depend on the Re or the VG shape. They also observed that the drag directly correlated with the *β*. The local *h*_m_ at the channel centerline increased sharply at the trailing edge of the VG for both delta wing and rectangular wing configurations. However, at a specific streamwise location, the local *h*_m_ for the DW was higher than that of the RW. For a delta wing with a *β* = 30° and a Re of 1815, the wing with an AR between 1.5 and 2 exhibited the best thermal performance. The study also showed that a single delta wing had the highest HT enhancement capability, followed by a DW, a pair of DW, an RW, and a rectangular wing.

In a study by Fiebig,^56^ significant lateral velocities, reaching up to 50% of the average axial values, were observed in a channel flow with an RW compared to a cross rib. The longitudinal vortices created by the winglet augment HT more effectively than the transverse vortices produced by the cross rib due to their superior ability to convey heat in the streamwise direction. The *f* for the cross rib rises by 286% compared to a plain channel, whereas, for the winglet configuration, it was less than half of the *f* experienced with the cross rib. In another study by Biswas *et al.*,^60^ it was observed that at a Re of 1580, increasing *β* of a single DW in a channel flow led to higher local maxima of spanwise average Nu near the trailing edge of the winglet due to stronger vortices at higher *β*. The performance of a single winglet was superior to that of a pair of winglets in terms of *j*/*f* values, with a *j*/*f* value of 0.95 observed for the channel with an AR = 5 at *β* = 15°.

Sarangi *et al.*^61^ researched wavy RWP to enhance HT in a fin-and-tube HX containing five rows of inline tubes. They explored the optimal wave height, length of the wavy winglets, and attack angle to achieve the highest *η*. Wavy RWVGs instead of plain RWP increased the temperature within the wake zone. This suggests that wavy RWP contribute to superior thermal mixing and enhances HT in the wake region. Additionally, they observed that positioning flat RWP over the second tube and wavy RWP over the third tube resulted in superior TEF.

**Fig. 6:**
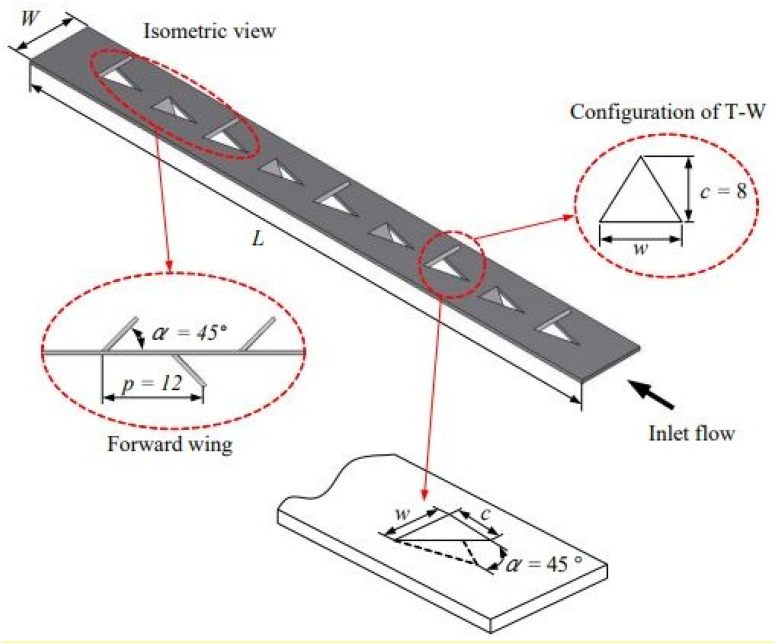
Geometries of the (a) longitudinal strip (L-S) insert and (b) double-sided delta-wing (T-W) tape insert. Reproduced with permission^63^ copyright © 2018 Elsevier.

In their research, Zheng *et al.*^62^ analyzed the aerodynamic and thermal characteristics of trapezoidal (TWVGs) and rectangular (RWVGs) winglets VG. Their findings demonstrated the superior performance of TWVGs in terms of HT. The study showed a maximum TEF of 1.756 at a Re of 543, highlighting the effectiveness of TWVGs in enhancing HT. Wijayanta *et al.*^63^ performed numerical analysis to examine the impact of double-sided delta-wings (DSDW) on HT and *f* characteristics (Fig. 6). A significant surge in Nu and *f* for tubes equipped with DSDW was noted compared to plain tubes, with enhancements of 1.77 and 11.6 times, respectively. Additionally, the findings demonstrated that incorporating DSDW could enhance heat performance (HP), with the highest TEF reaching 1.15. Zhang *et al.*^64^ piloted an experimental analysis of the thermal characteristics of novel V-shaped ribbed waveguide structures (V-RWVGs). They observed that the V-RWVGs produced multiple longitudinal vortexes, resulting in a significant increase in HT rate and *f* by 118% and 644%, respectively. In comparison to other configurations, the V-RWVGs exhibited superior heat performance.

Enhanced fluid mixing can be achieved through analysis of the VGs' geometry and placement, such as the number of VGs installed in the duct, as shown by Sun *et al.*,^65^ who studied the impact of varying numbers of RWVG on HT examined. They found that increasing winglets improved HT, with a maximum TEF of 1.27 observed. Awais and Arafat^66^ conducted a comprehensive numerical and experimental investigation into the influence of variations in the arrangement, position, and *β* of VGs on the flow's HT properties and Δ*p* distribution. Their findings revealed that including DWVG resulted in superior performance compared to RWVG. Oneissi *et al.*^67^ examined a newly designed inclined delta winglet and assessed its efficiency compared to a conventional DW. The innovative shape enhanced performance in reducing Δ*p* and enhancing VG. Khanjian *et al.*^68^ investigated varying *β* for a rectangular VG from 0 to 30°. The optimal angle was determined to be 25°.

**Fig. 7:**
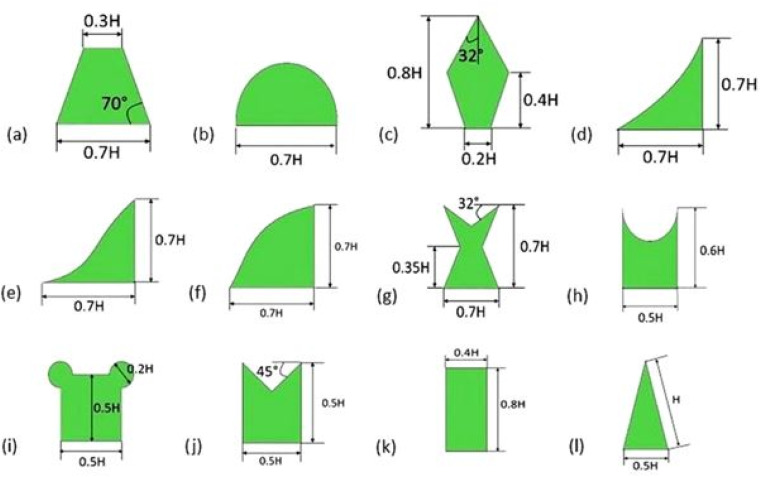
Geometric profile of the different wing type vortex generators (a) trapezoidal (b) semicircular (c) kite (d) parabolic (e) ogive (f) gothic (g) fish-tail (h) punched (i) bread (j) envelope (k) rectangular (l) delta wings. Reproduced with permission^69^ copyright © 2022 Elsevier.

Hasan *et al.*^69^ numerically explore how the cooling efficiency of Exhaust Gas Recirculation (EGR) systems can be enhanced by incorporating newly designed VG within the engine compartment of a vehicle. Twelve distinct shapes (Fig. 7) of proposed wing-type VG were examined numerically to evaluate the cooling impact of a single VG within the duct. A forward inclination angle of 135° was used across all studies, as this angle provides the optimal vorticity. The kite-shaped wing vortex generator demonstrated the most effective cooling performance among the various designs. Subsequently, these VG were arranged in various array configurations with five different pitch distances to identify the optimal pitch. With a 3 mm pitch distance, the gothic-type wing vortex generator outperformed the others, showing the best cooling performance and an 11% improvement in cooling compared to a single vortex generator. The impact of changing the exhaust gas inlet velocity on vorticity and cooling effectiveness was also evaluated. The study highlighted that the gothic-type wing VG was superior in reducing the outlet temperature, 12% more effective than discrete ribbed VG and 4% more effective than perforated louvred strip VG.

**Fig. 8:**
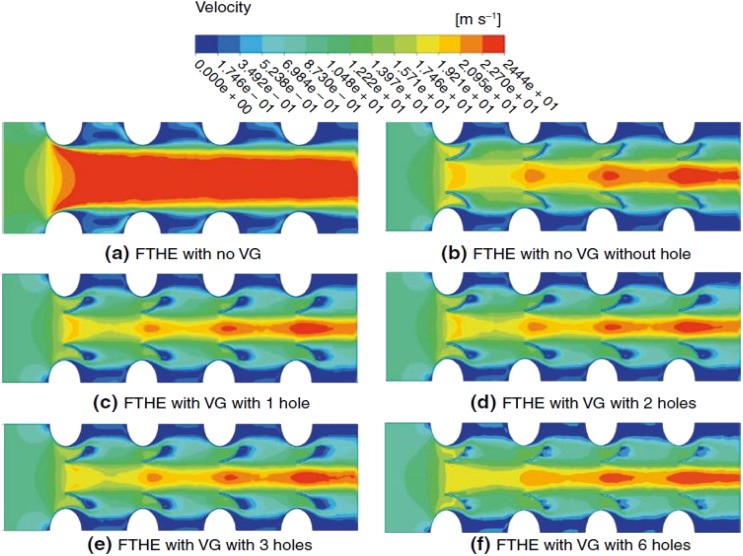
Velocity distribution of fin-and-tube HX with or without curved trapezoidal winglet for different cases at Re of 400. (a) FTHE with no VG, (b) FTHE with no VG without hole, (c) FTHE with VG with 1 hole, (d) FTHE with VG with 2 holes, (e) FTHE with VG with 3 holes, (f) FTHE with VG with 6 holes. Reproduced with permission^70^ copyright © 2023 Elsevier.

Saini *et al.*^70^ investigated the impact of curved TWVG with and without circular perforations. They varied the Re from 400 to 2000 while maintaining a *β* = 30° for the winglets. They tested configurations with 1, 2, 3, and 6 holes for the winglets with circular punched holes. The winglet without holes exhibited superior HT performance compared to the other configurations. However, the winglet with six circular holes showed minimal *f* and achieved the highest TEF [(*j*/*j*_0_)/(*f*/*f*_0_)^1/3^]. The study also noted that lower Re resulted in higher TEF. Fig. 5a illustrates the rise in dynamic energy as high-pressure airflow passes through the narrowest cross-sectional area between two tubes. Behind the tubes, a region of low-velocity wake forms. Introducing VGs creates a nozzle-like passage between the tube and VG, hindering air circulation. This passage converts pressure into kinetic energy, redirecting the high-momentum flow behind the tube region. Consequently, the size, layout, and positioning of VGs significantly reduce the wake area behind the tube. These factors considerably impact the local velocity characteristics within the flow field near the fin-and-tube HX, as depicted in Fig. 8b–f.

Enhanced HT consistently results in a rise in Δ*p*. Li *et al.*^71^ examined the influence of augmented Δ*p* on pumping power for enhancing HT by fitting a DWVG around the tube. They observed an improvement in THP when VGs were utilized around the fin and tube HX tubes. Promvonge and Skullong^72^ investigated the THP of V-shaped arrangements of rectangular and DWVGs. They determined that the V-shaped setup of the DWVG produced a slightly better TEF than the RWVG. To improve overall THP by reducing *f*, holes were punched into the surface of VG. Simulation results revealed the formation of jet flow when fluid passed through these holes, affecting the recirculation zone and eliminating stagnant fluid. This resulted in enhanced local HT and reduced *f*. The pitch of the multi-V-winglets VG played a critical role in influencing both HT and *f*, leading to an increase in the Nu of the smooth tube by 130.57–156.42%.^73^ Another study by Wijayanta *et al.*^74^ investigated the effects of punched DWVGs at different *β* on *f* and HT. The results showed a 264% increase in THP at an *β* = 70°, with an average *f* approximately 11.87 times higher than a plain tube. A few of the early works are mentioned in the Table 1.

**Table 1:** Few earlier research on vortex characterization on flat plates with VGs

Author	Type of VG	Parameter	Results
Ashill *et al.*,^75^ Ashill *et al.*^76^	Counter-rotating vanes forward wedge backward wedge single vane	*U*_∞_ = 10 to 40 m s^−1^, *δ* ∼ 60 mm, *h*/*∂* = 0.5, *e*/*h* = 10, *β* = ±14,^60^*β* = 10, 20, 30, 40, 50 (ref. 61)	Correlation of vortex strength against Re established, pitch among vanes of counter-rotating VGs reduces the mutual vortex interference
Yao *et al.*^77^	Single rectangular vane	*U*_∞_ = 34 m s^−1^, *δ* ∼ 35 mm, *h*/*∂* = 0.2, *e*/*h* = 7, *β* = 10, 16, 23	Detailed flow field data are obtained for a device-induced embedded streamwise vortex
Allan *et al.*^78^	Single trapezoid vane	*U*_∞_ = 34 m s^−1^, *δ* ∼ 45 mm, *h*/*∂* = 0.2, *e*/*h* = 7, *β* = 10, 23	CFD under estimated the peak vorticity by as much as 40% near the VG
Zhang and Wang^79^	RWP on the bottom wall	Re 500 to 7000, *β* = 20° to 40°, *h* = *H*/4 to *H*/2	Maximum HT performance is obtained when the *β* is 29°
Shyu and Jheng^80^	DWP, RWP, swept DWP (SDWP), and swept trapezoid WP (STWP)	200 < Re < 1000	SDWP showed max THP with Nu rise of 40% compared to other VGs
Syaiful *et al.*^81^	Perforated concave RW (PCRWs) and CRW	Re 3102 to 16 132	*h*_m_ of perforated CRW VG decreased by 1.02% compared to CRW, Δ*p* decreased by 15.38% for perforated CRW VG compared to CRW
Caliskan *et al.*^82^	Punched RVG (NPRVGs) and punched RVG (PRVGs)	Re (5181, 10 363, 20 328, 25 510), *β* = 15°, 30°, 45°, 75°, *b*/*e* = 0.04, 0.12, 0.2 and 0.36	The maximum TEF increased from 0.99 to 1.89 compared to NPRVGs
Mehra *et al.*^83^	DWVG of CFU and reverse CFU orientation	Spacing between winglets, laminar flow	An increase in TEF of 2.3% was noted compared to CFU
Santhanakrishnan *et al.*^84^	DWVG mounted on horizontal and vertical walls inside a PFHS	CFU and CFD, trailing edge gap length, laminar flow	PFHS with bottom plate-mounted VG and vertical plate fin-mounted VG are, respectively, 1.12 and 1.17 times higher than the baseline PFHS
Fahad *et al.*^85^	VG1 = three triangles on its top side, VG2 = concave shape arc, VG3 = rectangle and a triangle, VG4 = rectangle and two triangles at two corners, VG5 = rectangle with a triangular inside cut	Re = 4000–11 000, *β* = 30°, 60°, 90°, 120° and 150, *ψ* = 30°, 60°, 120° and 150° (horizontal rotation angle)	VG-1 gives the most increments, 38.2% in Nu and 80.38% in *f*, with a 1.63% rise in TPF
Dogan and Erzincan^86^	Novel VG	Re = 5000–25 000, transverse PR = 0.50, 0.25, 0.16, and 0.12, VG rows = 2, 4, 6, and 8	PR decreased, the Nu and *f* rose, with a maximum of 1.44 and 2.07 times higher in Nu and *f* at a transverse PR of 0.12, with the highest TEF value of 1.59 at the longitudinal PR of 1.5
Feng *et al.*^87^	Insertion-type (IT) longitudinal VG	Re of 414–1042	*f* and Nu in the minichannels with ITLVGs rises by 85.5–246.1% and 39.2–102.0%, PEC value of 1.50 at the Re of 1042
Ponmurugan *et al.*^88^	Rigid rectangular flapping longitudinal VG	Flapping frequencies (0.167, 0.25, and 0.5 Hz), Re = 3000–8000	Lowest flapping frequency 0.167 Hz yields highest HT augmentation of 38.54% and a 19.47% rise in *f* compared to a smooth channel

#### 3.1.2 VG in HX like fin-and-tube and other HX

The preceding investigation establishes that VG represents an exceptional supplementary flow manipulation technique for augmenting HT. Concerning the passive vortex generators, their straightforward manufacturing process and the lack of requirement for an external power source to actuate them confer even greater advantages. Due to these benefits, the utilization of VGs extends beyond a single type of HX, finding widespread application.

**Fig. 9:**
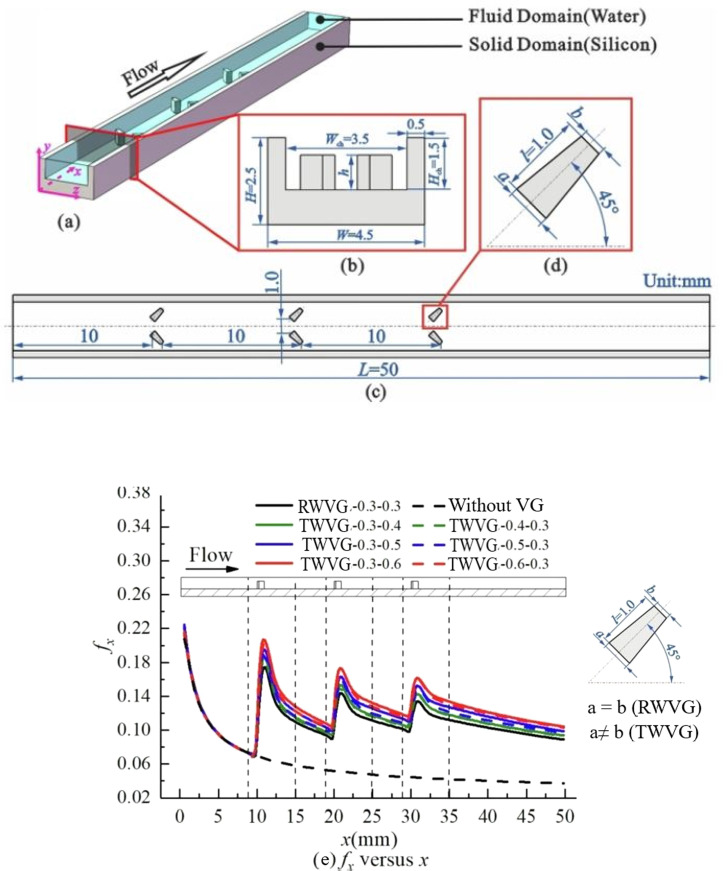
Schematic diagrams of (a) the computational domain of mini-channel, (b) sectional side-view, (c) top-view and (d) TWVG.^62^ (e) The deviations in local fanning friction factor along the flow direction for MCW with trapazoidal VG and smooth micro channel. Reproduced with permission^62^ copyright © 2022 Elsevier.

However, a significant drawback accompanying HT augmentation *via* vortices is the accompanying Δ*p* losses, as reported by numerous authors.^56–88^ The primary constituent of these losses is the Δ*p* in the form of drag resulting from the Δ*p* discrepancy around the vortices along the flow direction. This pressure imbalance translates into forces acting in opposite directions on the fluid particles in the main flow. Consequently, the retardation or momentum loss experienced by the fluid particles necessitates compensation through additional pumping force, which leads to elevated operating costs. From Fig. 9, it is evident that the improvements in flow resistance in the microchannel with longitudinal trapazoidal VG (TWVGs) are influenced by the geometry of the VG.

Recently, numerous researchers have devised novel methodologies to address the Δ*p* inherent to VG. These approaches encompass alterations to the shape of classic VG and innovative VG designs aimed at reducing *f* losses or enhancing the overall PEC, thereby realizing greater HT with the same Δ*p*. A few novel VG shapes and orientations have been discussed below.

The evaluation of THP often necessitates precise quantification of flow rate, Δ*p*, and temperature. The experimental methodologies employed to investigate the THP of HT surfaces on the air side encompass techniques such as laser light sheet illumination (LLS), naphthalene sublimation technique, infrared thermography (IT), laser Doppler velocimetry (LDV), liquid crystal thermography (LCT), hot-wire anemometry (HWA), and particle image velocimetry (PIV). Tables 2 and 3 present a chronological overview of representative experimental techniques for measuring flow rate, pressure, and temperature.

**Table 2:** Types and performance features of VGs in a duct/flat plate

Author	Type of VG	Re range	Methodology used	Objective
Garimella and Eibeck^89^	Horizontal channel with two different half-delta wings	700–5200	30 heated copper elements transfer heat to HX mounted on the detachable hatch	HT augmentation, Δ*p*
Fiebig *et al.*^55^	DW and RW on a flat plate	1360–2270	Flow visualization was acquired using LLS, unsteady LCT determines local *h*_m_	Flow pattern, *h*_m_, drag coefficient, *j*-factor, TEF
Tiggelbeck *et al.*^90,91^	Single and double rows of half-DW on a flat plate	1600–8000	PIV with glycerine captures for flow structure, LLS is for flow field visualization, LCT measures local *h*_m_	Flow path, vortex property, local Nu and *C*_d_
Tiggelbeck *et al.*^92^	DW, RW, DWPs and RWPs on a flat plate	2000–9000	LCT is used to acquire the *h*_m_ on the wall	Local Nu number
Gentry and Jacobi^93^	DW on a flat plate	600, 800, 1000	LLS for flow path, naphthalene sublimation for *h*_m_	*S*_h_, *C*_d_
Kotcioğlu *et al.*^94^	RWPs on a flat plate	Turbulent	Smoke generator using Hele–Shaw apparatus	Flow path, average Nu and *f*
Gentry and Jacobi^95^	DW on a flat plate	300–2000	LLS flow contour, PIV to provide *h*_m_, vane-type vortex meter to capture flow	Flow path, *S*_h_ distribution, average *S*_h_, TEF, Δ*p* penalty
Yuan *et al.*^96^	RWPs on a flat plate	5000–47 000	25 rows of copper-constantan thermocouples mounted uniformly in the flow direction to measure local *h*_m_	Local and average Nu and *f*, with correlations
Leu *et al.*^97^	RWPs in a finned circular-tube HX	400–3000	The dye injection technique IT is used for flow visualization, and HWA captures inlet velocity	Temperature and Nu distribution, average *h*_m_ and Δ*p*
Chen and Shu^98^	DW on a flat plate	4430–11 820	LDV for flow structures and velocity, 25 thermocouples acquire surface temperature	Flow structure and avg Nu distribution
Wang *et al.*^99^	RWPs on a flat plate	3000–20 000	Deionized water as a working fluid	Local and Avg Nu, with correlations
Hernon and Patten^100^	DWPs on a flat plate		HWA acquire velocities	Local velocity, BL thickness
Yang *et al.*^101^	DW, semi-circular wings, TW, dimple wings on a flat plate	120–600	Multiple nozzle code testers capture air flow rate	Average *h*_m_ and Δ*p*, *j*-factor, *f*, inverse Graetz number
Promvonge *et al.*^102,103^	Combined ribs and winglets in a triangular ribbed channel	5000–22 000	Multiple nozzle code testers acquire airflow rate	Average Nu and *f*, PEC
Min *et al.*^104^	Modified RWPs on a flat plate	5000–17 500	HWA, PIV generate glycerol particles, 54 thermocouples capture temperature distribution	Average Nu and *f*, local Nu number, secondary flow vector distribution
Wu and Tao^105^	DW and punched holes on a flat plate	500–2000	Eight thermocouples at the inlet and 8 at the outlet measure air at the inlet and outlet, and 16 thermocouples measure surface temperature	Temperature distribution, average Nu
Aris *et al.*^106^	DW on a flat plate	1573–3712	Uniform temperature condition a ng PIV measurement	Local Nu number, average Nu number and apparent *f*
Zhou and Ye^107^	RWPs, TWPs, DWPs, and CTWPs on a flat plate	700–26 800	24 T-type thermocouples acquire temperature distribution, with 21 T-type thermocouples measuring surface temperature	Average Nu and *f*
Zhou and Feng^108^	RWPs, TWPs, DWPs, CTWPs with or without holes on the winglet on a flat plate	650–21 000	24 T-type thermocouples acquire temperature distribution at the inlet and outlet, with 21 thermocouples measuring surface temperature	Average Nu and *f*

**Table 3:** Performance features of VG in fin and tube HX

Author	Type of VG	Re	Methodology	Measurement
Fiebig *et al.*^109^	DWPs in HX (circular tube)	600–2700	HWA (velocity distribution), LLS to Nu distribution	Local and Avg Nu and *f*
Yoo *et al.*^110^	RWPs in HX (circular tube)	800–4500	PIV and mass transfer, analogy equation to calculate *h*_m_	Local and Avg Nu with *f* distribution
Torii *et al.*^111–113^	DWPs in HX (circular tube)	350–2100	Stainless steel heating ribbon for uniform heat flux	*j* and *f*-factor
O'Brien *et al.*^114^	DWPs in oval-tube HX	600–6500	IT for temperature distribution with a mass flow meter to capture airflow rate	*h*_m_, Nu and *f* distribution
Sommers and Jacobi^115^	Delta wings HX (circular tube)	500–1300	An upstream cooling coil and a controlled steam injection system to control the temperature and humidity of the air, HWA acquire airflow velocity	TEF, Δ*p* penalty, *j* and *f*, *j*/*f*, average frost density with time
Pesteei *et al.*^116^	DWPs in parallel plates with a single cylindrical obstacle	2250	23 thermocouples to measure surface temperature	Local *h*_m_, average Nu and *f*
Shi *et al.*^117^	DWPs in HX (flat tube)	<3000	LCT (naphthalene) for heat–mass transfer analogy	Local and Avg Nu and *f*, *j*/*f*
Allison and Dally^118^	DWPs in HX (circular tube)	2600–4600	DIT and IT for the flow visualization	*j*, *f* and *j*/*f*
Joardar and Jacobi^119^	DWPs in HX (circular tube)	220–960	Stem is injected into a centrifugal mixer and mixed with air for uniform temperature and humidity	Avg *h*_m_*j*, *f*, *j*/*f*
Tang *et al.*^120,121^	DWPs and mixed fins HX (circular tube)	4000–10 000	Steam-air heat exchange, pitot tube measures velocity	Avg Nu *j*, *f*, *j*/*f*, factor, PEC with Nu and *f* correlations
Aris *et al.*^122^	DW in a finned HX (circular tube)	330–960	Orifice plate acquires flow rate	Avg Nu and *f*
Wu *et al.*^123^	DWPs in HX (circular tube)		Flow meter measures velocity	Avg *h*_m_ and Δ*p*
Wang *et al.*^124^	Semi-dimple winglet pairs in plain or louvre-finned HX (circular tube)		Air flowrate captured using multiple nozzle	*h*_m_ and Δ*p*
Abdelatief *et al.*^125^	RWPs in a wing-shaped-tubes-bundle HX	1850–9700	8 thermocouples measure surface temperature	Avg Nu and *C*_d_

The preceding section demonstrates that VG is an exceptional secondary flow manipulation tool for enhancing heat transfer. In the case of passive VG, their straightforward manufacturing process and the absence of a requirement for an external power source to actuate them make them even more advantageous. Due to these benefits, the utilization of VG is not limited to a specific type of HX; instead, their application is widespread.

The principal disadvantage linked to heat transfer amplification *via* vortices is the rise in Δ*p*, as documented by various preceding investigations,^60–77^ whose primary element is Δ*p* differential/form drag, which arises as a consequence of the pressure difference around vortices along the direction of the primary flow. This pressure imbalance results in a force acting upon the fluid particles in a direction counter to the main flow. The deceleration or momentum deficit experienced by the fluid particles must then be offset by an additional pumping force, which ultimately corresponds to higher operating costs.

Recently, numerous researchers have developed innovative methodologies to report the problem of Δ*p* inherent to VG. These approaches include modifying the profile of classic VG^78–97^ and introducing novel VG designs to reduce *f* or increase the overall PEC, *i.e.*, achieving greater HT with the same Δ*p*. A few other novel techniques deal with unavoidable Δ*p* by perforating the VGs^69,81,104,108,126^ or by using curved VGs^70,107,127^ and rigid/flexible VGs.^87,128^

A fin-and-tube HT typically consists of a tube through which a liquid flows and a surrounding space where a gaseous medium circulates. The *h*_m_ on the liquid side is significantly higher than on the gas side, indicating a difference in thermal resistance between the two sides. Fins are commonly used to enhance HT on the gas side by increasing the HT area, but their efficiency can limit this improvement.^129^ Using VG on the gas side can lead to substantial gains in HT. The recirculation region and BL separation can be effectively managed by strategically placing VG, particularly behind the tube, reducing low- HT areas.^130^ Studies have shown that VG can enhance HT in various types of HX, including fin-and-tube configurations, as well as in applications such as circular pipes, heat sinks, and micro and nanofluid systems. Their potential benefits extend to various HX systems, from refrigeration units to SAH.^131–136^

**Fig. 10:**
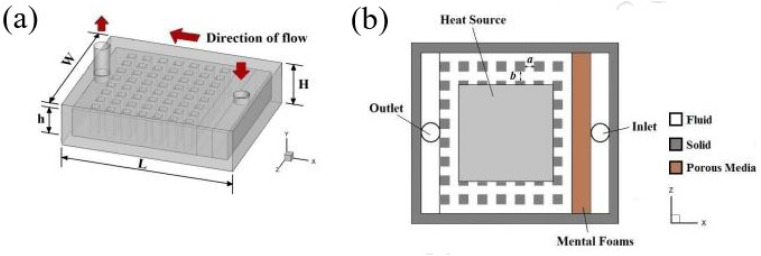
Structure diagram of micro square pin-fin heat sinks (a) external view. (b) Bottom view. Reproduced with permission^137^ copyright © 2015 Elsevier.

Zhao *et al.*^137^ numerically simulated the flow and heat transfer characteristics, leading to an optimized design for the micro square pin-fin heat sink of varying pin-fin porosity and the angle at which the pin-fins are positioned (seen in Fig. 10). A comparison was made between square pin-fins and column pin-fins to highlight the features and benefits of each design. The numerical results revealed that both pin-fin porosity and positioning angle significantly impact the micro square pin-fin heat sink's cooling capacity and thermal performance. The optimal porosity and angle for maximum thermal performance were 0.75 and 30°, respectively.

Additionally, micro heat sinks with optimized square pin-fins demonstrated superior thermal performance compared to those with column pin-fins. This suggests that micro square pin-fin heat sinks hold significant potential for improving thermal management in high-energy-density electronic devices.

**Fig. 11:**
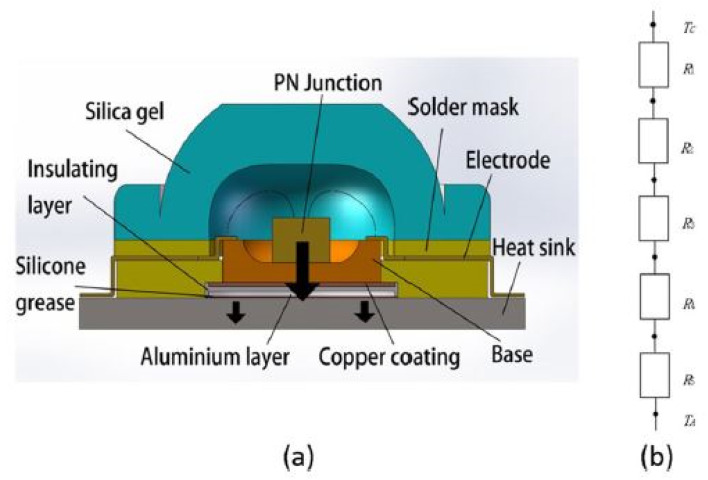
(a) LED cooling system and (b) its corresponding thermal resistance network. Reproduced with permission^138^ copyright © 2022 Springer Nature.

Xiang *et al.*^138^ experimentally and numerically first developed a theoretical model to calculate the thermal resistance of micro-channel heat sinks (Fig. 11). Following this, the fabrication process for these heat sinks was detailed. The heat transfer performance of the fabricated micro-channel heat sink was then evaluated using a specialized testing platform.

The results demonstrated that the micro-channel heat sink significantly outperforms traditional metal solid heat sinks regarding heat dissipation and is highly suitable for high-power LED applications. Additionally, the micro-channel structures within the heat sink were optimized through an orthogonal testing approach. This optimization process led to further improvements in the heat dissipation performance of the micro-channel radiator. A few additional works done are shown in Table 3.

### 3.2 Effect of geometry parameters

The numerical investigation conducted by Lei *et al.*^139^ explored the influences of the *β* from 10°–50° and the *Λ* spanning 1–4 of DWPs on the HT and Δ*p* characteristics of a fin-tube HX, across a Re domain of 600–2600. The results revealed that both the HT coefficient and the *f* escalated with a surge in the angle *β*, although the trend was less pronounced for HT than the gradually strengthening influence on Δ*p*. The *j*/*f* diminished with a rise in the Re for all the enhanced HX. The DWPs with an angle *β* of 20° and *Λ* = 2 delivered optimal performance for the given range of Re. For this configuration, the *j* experienced an enhancement of 35.1–45.2%, while the corresponding *f* increased by 19.3–34.5%.

**Fig. 12:**
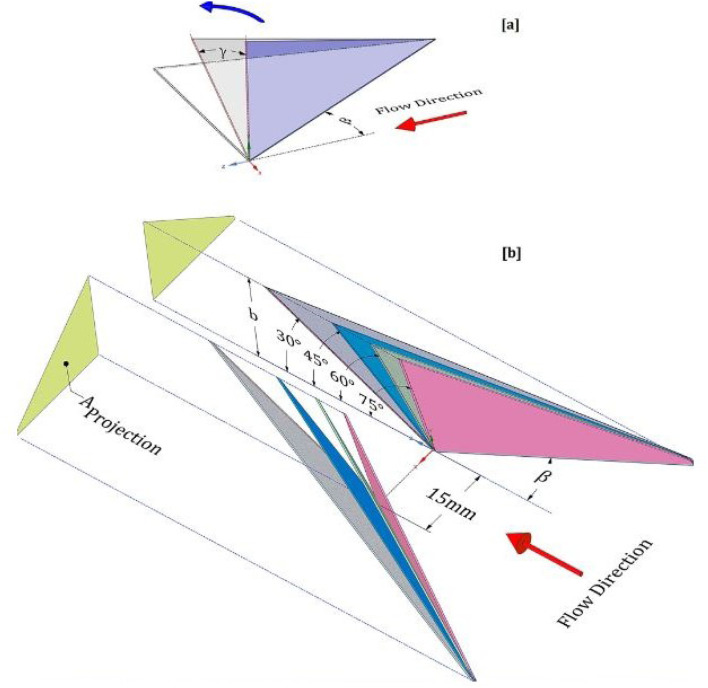
Schematic of inclined DW. (a) Conventional approach. (b) Innovative approach. Reproduced with permission^141^ copyright © 2024 Elsevier.

Pham *et al.*^140^ worked on flexible VG capable of generating swirl flow and compared it to static VG. A self-fluttering VG, machine learning played a pivotal role in analyzing extensive experimental data encompassing diverse design parameters. The aim was to identify a universal criterion governing the onset of large oscillatory motion and its impact on thermal transport and frictional pressure loss characteristics. Additionally, this study compared the thermal-hydraulic efficiencies of the flapping vortex generator with a collection of traditional ones of similar configurations. Demirağ *et al.*^141^ numerically studied novel inclined DW impacts on HT and *f* characteristics across a Re range of 5000–17 500. The investigation focuses on evaluating the effects of various geometric and arrangement parameters of inclined DW, including attack angles (*β* = 30°, 45°, 60°), inclination angles (*α* = 30°, 45°, 60°, 75°), and transverse PR (Pt = 0.166 and Pt = 0.333). The configuration is shown in Fig. 12. The results indicate that novel DW with lower *α* notably reduces the *f*. Significant increases in TEF values are observed, reaching 6.09% at Re = 5000 and 9.80% at Re = 17 500 for Pt = 0.333 compared to the reference case of DW. Moreover, the highest TEF value of 1.33 is achieved with inclined DW at *α* = 30°, *β* = 30°, and Pt = 0.166.

Leu *et al.*^142^ study the consequences of the *β* of block-shape VGs in plate-fin and tube HX, for Re between 400 and 3000. The inclined block-shape VGs with a *β* = 45° arrangement provide maximum TEF across all Re ranges, *j* augmented by 8–30% with *f* by 11–15%.

The investigation conducted by Jang *et al.*^143^ explored the effects of *β* of VGs ranging from 30°–60°, as well as the VG spanwise spacing (Ly) between 2 to 20 mm, on the TEF of a fin-tube HX-equipped with block-type VGs. The findings for both the inline and staggered VG configurations revealed that increasing the values of *β* and Ly intensified the strength of the longitudinal vortices generated, leading to enhancements in both the *j*-factor and the *f*. Interestingly, the inline VG arrangement was more effective in improving HT than the staggered arrangement. The estimated area reduction ratio ranges were 14.9–25.5% for the inline configuration and 7.9–13.6% for the staggered configuration across the Re range of 400–12001
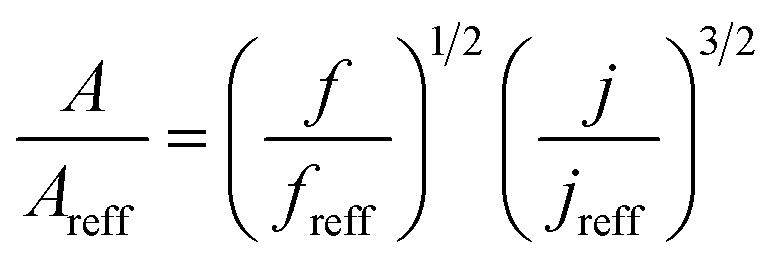


The research conducted by Gentry and Jacobi^144^ presented the concept of local and average *Ω* to capture the interaction between the vortex and the BL for the evaluation of DWVGs, with local and average. The local *Ω* determined using eqn (2) and (3)

2 *Ω* = Pef(∂*) where *f*(∂*) = ∂*^5/2^ exp(1 − ∂*^5/2^) with ∂* = *∂*_c_/*∂*_b_

where *δ*_b_ = boundary layer thickness, *δ*_c_ is core to plate distance3
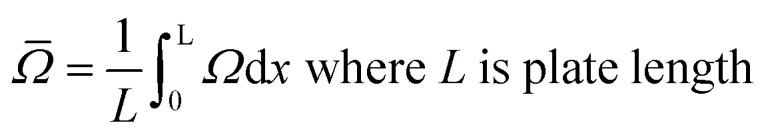


The findings revealed that the vortex strength increased with the *Re*, *Λ*, and the *β*, but its strength decayed downstream. In sections where the vortex-induced a surface-normal inflow, the local *h*_m_ could increase up to 3 times the baseline flow. For Re be 300–2000, the DWVGs resulted in a 50–60% augmentation in *h*_m_ and two times higher in Δ*p* for flat plate (Table 4).

**Table 4:** Empirical correlation based on the application of VG

Authors	VG type	Equation	Parameter
Kotcioğlu *et al.*^145^	RWPs in rectangular channel	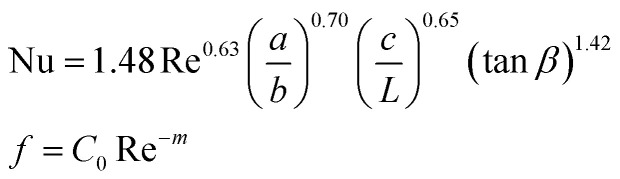	(*a*), duct height (*b*), streamwise winglet length (*c*), and *β*, Pr = 0.71 and *β* = 0–27°, Re = 3000–30 000
Wu *et al.*^146^	DWPs in a fin and tube HX	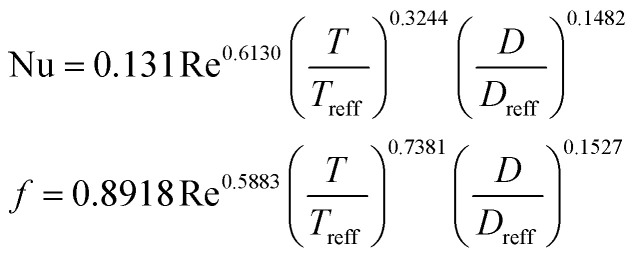	Reynolds number range of 500 to 4200, fin collar outside diameter (*D*), and fin pitch (*T*)
	Curved DWP in a fin and tube HX	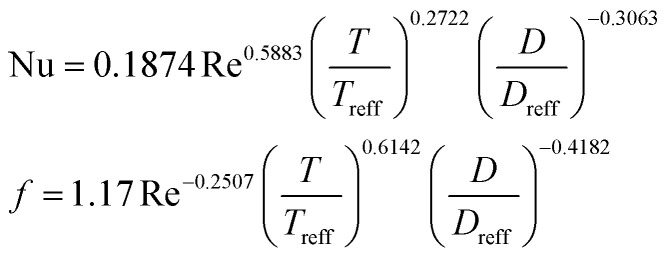	
Zdanski *et al.*^147^	Punched DEGs over tube bank	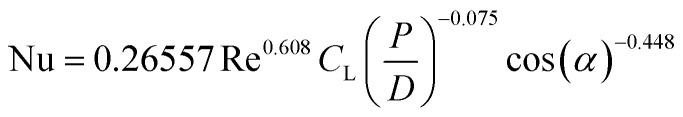	PR = 1.28–2.31 Nu rise by 30% and Δ*p* by 40%
Gururatana *et al.*^148^	NACA0024 VG	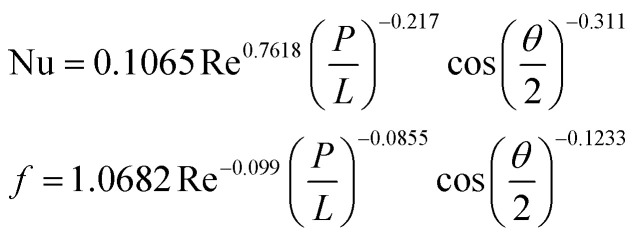	*β* = 60°–120°, Re = 3923 to 19 617, *P*/*L* = 3–7, VG = 90° and *P*/*L* = 4 show a maximum TEF of 1.62 at lower Re of 3923
Yaningsih *et al.*^149^	Double-sided DWT VGs	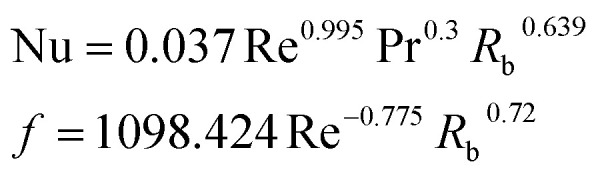	Re ranging from 5500 to 14 500, *R*_b_ at 0.28, 0.35, and 0.42, Pr = 0.2, DWTs increased Nu by 364.3% and *f* by 15.5 times
Lofti *et al.*^150,151^	Smooth wavy fin-tube HX with four recently proposed VGs, ARW, CARW, RTW	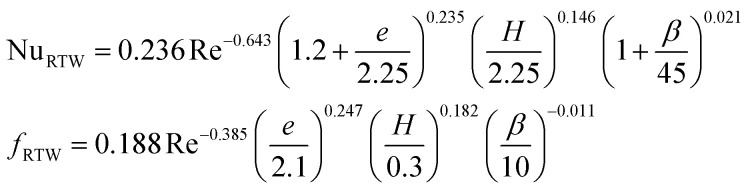	Re 500–3000, 15° ≤ *α* ≤ 75°, 0.65 ≤ *e* ≤ 1, and 0.8 mm ≤ *H* ≤ 1.6 mm
		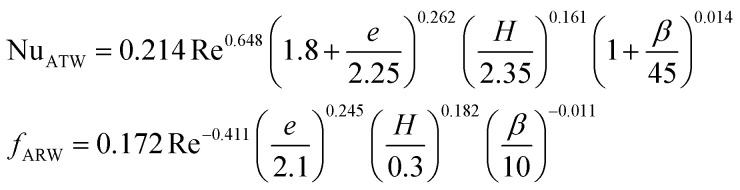	
		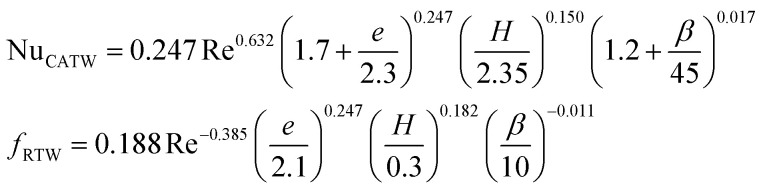	
Koolnapadol *et al.*^152^	SAH with RWVG	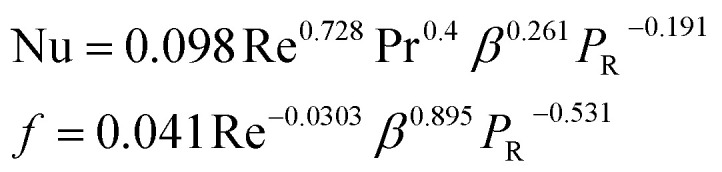	HT rate for using RWVG is 5.79 times while *f* is 43.97 times at *β* = 60°, PR = 1.0

## 4. Surface depressions/protrusions for HT augmentation

In recent years, the incorporation of dimple or protrusion structures has gained increasing attention in HT equipment to intensify HT. Ligrani *et al.*^153^ tested the HT properties of a plate HX featuring top protrusions and bottom dimples and a smooth top and bottom dimples configuration. Their study concluded that the HT capacity of the model with top protrusions and bottom dimples was larger than that of smooth top and bottom dimples under turbulent flow conditions.

Surface irregularities, including depressions or cavities like dimples and convex protrusions such as ribs, represent another passive technique for generating secondary flows to enhance heat transfer. As predicted by Göertler's research, the flow behaviour over a concave surface resembles the Taylor vortices formed within the annular space between two rotating cylinders (concentric).^154^ This VG on a concave surface is commonly referred to as Taylor–Göertler vortices. The existence of a pressure differential across the radial axis of the concave surface induces flow irregularities within the fluid medium. Subjected to centrifugal forces, these perturbations generate counter-rotating vortical structures, which progressively amplify along the downstream direction.^155,156^ The stability characteristics, as well as the transition from laminar to turbulent BL or the critical Re are determined by the Göertler number and concave surface curvature.^157,158^

The pioneering implementations of surface irregularities were primarily identified within the domain of aerodynamics, especially for BL management and drag diminution. For instance, concave indentations akin to dimples have been observed to reduce the drag coefficient of golf balls (Bearman and Harvey^159^) as well as circular cylinders (Bearman and Harvey^160^). These surface depressions have also been noted to delay circular cylinders' separation point, as Kimura and Tsutahara documented.^161^ The depressions on a surface, such as those found on a golf ball, mitigate pressure drag by triggering the transition of the laminar BL into a turbulent state.

The dimensional scale of a surface imperfection relative to the boundary layer thickness governs whether it will incite perturbations within the boundary layer flow or the primary flow field. Protrusions can be classified into two distinct categories: 2D and 3D. A 2D protrusion exhibits a continuous length along the spanwise direction, whereas a 3D protrusion is discontinuous or discrete, as depicted. In the case of a 3D protrusion, the streamwise vorticity component induces vortex stretching in the primary flow direction, an effect that is not observed for 2D protrusions.^162^

Regardless of the boundary layer state, whether laminar or turbulent, the general flow field around a surface irregularity exhibits a qualitatively analogous pattern. This includes the formation of vortical structures resembling horseshoes around the disturbance, which are then convected downstream as longitudinal vortices, the recirculation of the upstream separated flow, and the presence of vortical features in the wake region. The longitudinal vortices can persist for hundreds of multiples of the protrusion height in the downstream direction, and they are more heavily influenced by the protrusion's location and height rather than its specific geometry.^163^ Moreover, the secondary flow in the form of longitudinal vortices, combined with the increase in flow turbulence induced by dimples and protrusions, leads to enhanced fluid mixing and thinning of the BL, ultimately resulting in improved HT performance.

**Fig. 13:**
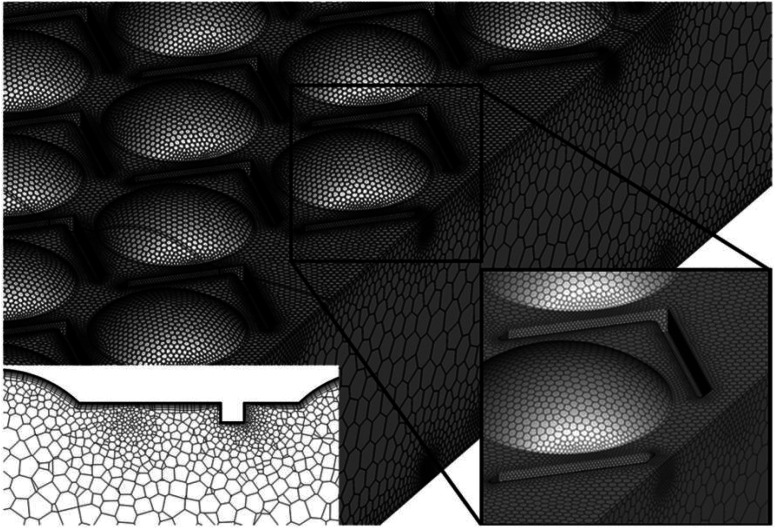
The geometry of deep dimple tube. Reproduced with permission^166^ copyright © 2023 Elsevier Masson SAS.

**Fig. 14:**
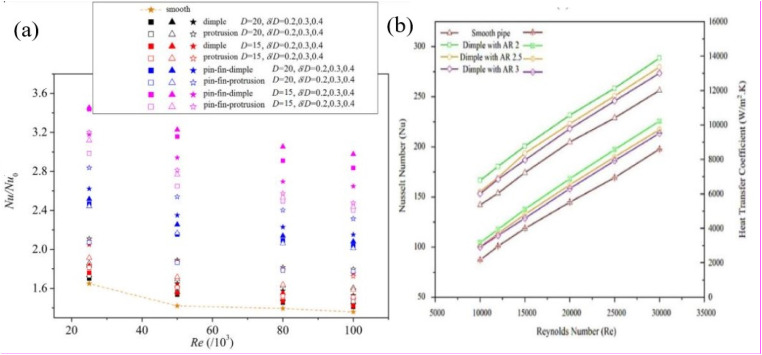
Variation of Nusselt number and convective heat transfer coefficient with Reynolds number. (a) Summary of average Nu/Nu_0_ in each channel. Reproduced with permission^167^ copyright © 2016 Elsevier. (b) Using 1% Al_2_O_3_/water nanofluid for different geometries. Reproduced with permission^168^ copyright © 2023 Elsevier.

Paul *et al.*^164^ investigated a heat transfer wall's flow and HT performance arranged with dimples. They pointed out that the dimples created stronger vortices, thus enhancing fluid turbulence, thinning the boundary layer, and intensifying the heat transfer. Xu *et al.*^165^ introduced protrusions in a heat transfer channel and experimentally studied the channel's heat transfer and flow properties with different arrangements and numbers of protrusions. The study concluded that the average Nusselt number was the highest when the protrusions were arranged along the transverse direction, and the number of protrusions was three. Combining dimples or protrusions with other structures has also proven to be an efficient way of intensifying heat transfer. Zhang *et al.*^166^ proposed a novel contraction channel mounted with V-shaped ribs and dimples (Fig. 13). The study pointed out that this hybrid structure increased the fluid turbulence and produced stronger longitudinal vortices, further enhancing heat transfer. Xie *et al.*^167^ arranged a combination of pin fins and dimple protrusions in the turning channel of a turbine blade. Their study noted that the temperature of the cooling channel surface decreased significantly after the dimple protrusions and pin fins were combined, and the friction coefficient increased slightly compared to the smooth cooling channel (Fig. 14a). Geometrical factors, such as the depth and shape of protrusions or dimples, greatly influence the thermal characteristics of HX channels. Ahmad *et al.*^168^ investigated the impact of nanofluids on spherical double-dimpled surfaces regarding HT and Δ*p* characteristics. This encompassed analyzing *h*_m_, temperature distributions, Nu, Δ*p*, and *f* across various operational scenarios. The corrugated profile of a double-dimpled pipe notably enhances thermal performance compared to a straight pipe profile (Fig. 14b). Their findings indicated that the double-dimpled configuration yielded a 20–25% higher *h*_m_ than the smooth pipe. Jang *et al.*^169^ combined ribs with oval and spherical protrusions and experimentally studied the effect of dissimilar protrusion shapes on the TEF in an HT channel.

**Fig. 15:**
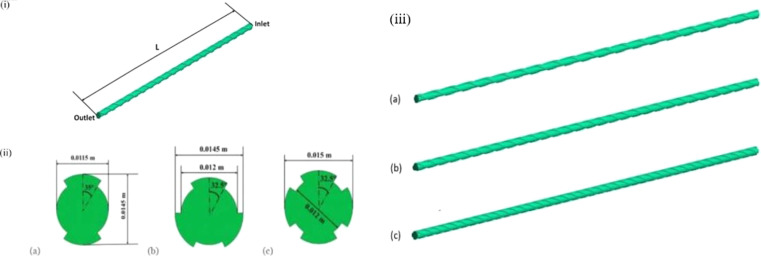
(i) 3D model of a straight tube HX and (ii) the cross-sectional profile (iii) isometric view of (a) 2 rib (b) 3 rib (c) 4 rib heat exchangers. Reproduced with permission^170^ copyright © 2023 Elsevier.

Hasan and Bhuiyan^170^ used numerical methods to investigate how different ribbed geometries, combined with varying coil revolutions, affect the performance of a straight heat exchanger. The research considered three rib configurations (2 ribs, 3 ribs, and 4 ribs) as seen in Fig. 15 and three coil revolution counts (10 revolutions, 20 revolutions, and 30 revolutions), resulting in nine different setups. Air was used as the working fluid under heating conditions. The study analyzed each configuration's velocity and temperature profiles, local Nu, Δ*p*, *f*, and entropy generation. Results indicated that HT performance improved with fewer rib heads and higher coil revolutions. Specifically, the configuration with 2 ribs and 30 revolutions achieved the highest Nu, while the configuration with 4 ribs and 10 revolutions had the lowest. Additionally, *f* increased with higher coil revolutions for a given rib head count. Notably, the *f* for the 4-rib configuration was twice as high at 30 revolutions compared to 10 revolutions. Conversely, the 4-rib, 10-revolution configuration exhibited the lowest entropy generation among all configurations.

Burgess and Ligrani^171^ and Won *et al.*^172^ examined the consequences of three different protrusion depths. They found that the Nu increased with the protrusion depth. Rao *et al.*^173^ studied the *f* and Nu of three types of dimples with depth-to-diameter ratios of 0.2, 0.1, and 0.067. They pointed out that the minimum value of the comprehensive PEC occurred when the depth-to-diameter ratio was 0.2 due to excessive Δ*p*. Briclot *et al.*^174^ explored the performance of Al_2_O_3_ nanofluid within a basic tubular setup under both laminar and transient flow conditions. Their study, which covered Reynolds numbers from 500 to 4500 and nanofluid concentrations between 0.75% and 5%, found that the nanofluid exhibited lower thermal efficiency compared to distilled water during transient conditions. Additionally, variations in nanofluid concentration did not affect the critical Re. In another study, Zhang *et al.*^175^ conducted both numerical and experimental investigations of a hybrid CuO–Al_2_O_3_ nanofluid with mass concentrations ranging from 1% to 3% in turbulent flow conditions. They compared various numerical methods with experimental data, revealing that the performance enhancement coefficient (PEC) increased with higher nanofluid concentrations but decreased with higher Reynolds numbers, with PEC values ranging from 1.01 to 1.29. Shi *et al.*^176^ pointed out that incorporating dimple or protrusion structures within turbine blades can significantly enhance cooling.

Similarly, Kong *et al.*^177^ numerically explored the effect of dimple-protrusion density on the thermal-hydraulic characteristics of a rectangular channel, concluding that increasing the density of these surface features was beneficial for improving HT performance. Wang *et al.*^178^ discovered that arranging protrusions on solar air absorber panels (Fig. 15) can remarkably improve the efficiency of solar energy utilization. Meanwhile, Silva *et al.*^179^ investigated the thermo-hydraulic properties of a channel fitted with dimples, proposing that in contrast to a flat plate, the HT capacity of the dimpled channel was approximately 2.5 times greater. The combined use of dimples and protrusions has been shown to have notable effects on HT and flow losses within a channel. These numerical studies afford treasured insights into the performance characteristics of these surface modifications to enhance HT.

**Fig. 16:**
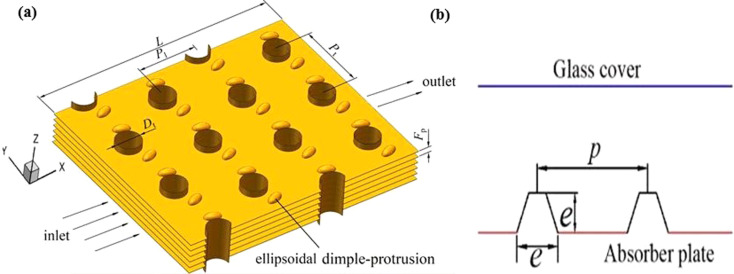
Protusion used in HX. (a) Elleptical dimple.^180^ (b) Frustum-shaped. Reproduced with permission^178^ copyright © 2023 International Solar Energy Society.

Song *et al.*^180^ numerically examined HT characteristics of circular tube-fin HX on the fin side by introducing ellipsoidal dimple-protrusions on the fin side of different *β* (0°–40°). The ellipsoidal dimple-protrusions substantially increase both secondary flow intensity and HT capability (Fig. 16). Compared to smooth channels and channels equipped with VG, the secondary flow intensity increases by up to 78.62% and 41.57%, respectively. In comparison, Nu increases by a maximum of 29.01% and 19.03% across the Re range of 1500–5000. The TPF reaches a maximum of 1.161, marking a 16.1% improvement over smooth channels.

Xie *et al.*^181^ inspected the thermo-hydraulic properties of a tube with helical dimples and proposed that this design led to a 120% to 270% increase in heat transfer. Xie *et al.*^182^ introduced a protuberant tube that can generate strong longitudinal vortices, increase fluid disruption, and disrupt the thermal BL, ultimately improving HT. They also studied the geometric parameters of the protrusions to determine the optimal arrangement for the highest HT.

Zhang *et al.*^183^ proposed a novel cross-combined ellipsoidal dimple tube and concluded that its heat exchange capacity was improved by 18.8% to 48.3% compared to a single ellipsoidal dimple tube. Zheng^184^ and Rad *et al.*^185^ suggested that the protrusion and dimple structure can disrupt the thermal BL, promote fluid mixing, and ultimately enhance the HT between the fluid and the wall surface of the tube. In a further study,^186^ applying ellipsoidal protrusions on the bottom fin of channels in a tube-fin HX resulted in apparent improvements in HT. However, that study only considered the top fin of the channel as a plain fin. In practical applications of tube-fin HX, the protrusions are stamped out from the fin surface, and the protrusions in one channel also act as dimples in the adjacent channel. Therefore, the combined effects of dimples and protrusions should be considered in the tube-fin HX channel.

**Fig. 17:**
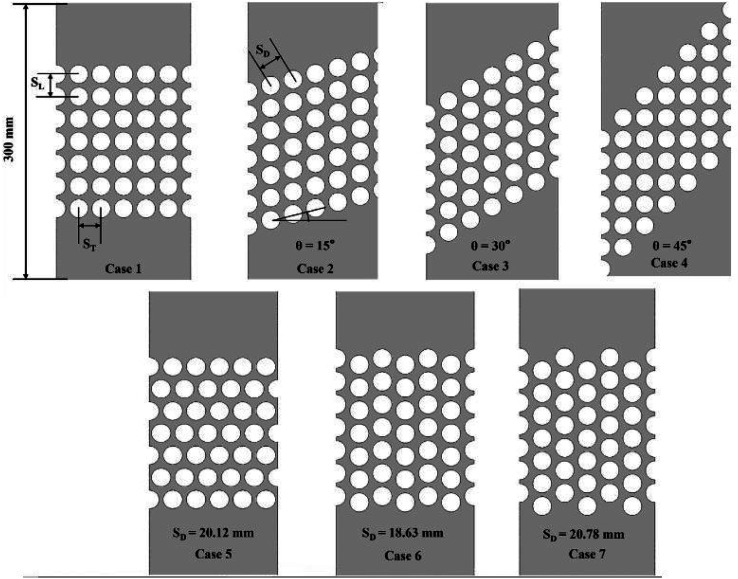
Schematic representation of the tube bundle layout. Reproduced with permission^187^ copyright © 2024 Elsevier.

Nuhash *et al.*^187^ numerically examined thermal and frictional characteristics of Lead–Bismuth Eutectic (LBE) crossflow over tube bundles on the shell side of a Helical-coiled Once-Through Steam Generator (H-OTSG). The model examines configurations, including inline, obliquely staggered, triangular, and rotated square arrangements. The k-ω SST turbulence model, paired with Kays' turbulent Pr model, was employed for this analysis. The arrangement of tube bundles (Fig. 17) notably affects the development of transverse flow, vortical structures, and the heat transfer rate within the flow domain. The triangular configuration yielded the highest Nu of 8.2, reflecting a significant crossflow effect and a 10% improvement over the inline configuration, which had a Nu of 7.45. However, the triangular layout also showed a higher *f* of 0.45, a 20% increase compared to the *f* observed in the inline setup. Increasing the oblique angle reduces both the HT rate and *f*. At a 45° oblique angle, the turbulence intensity results in a Nu of 7.05, which is higher than the 6.93 observed at a 30° angle but lower than the 7.15 observed at a 15° angle. For rotated square configurations, the Nu decreases, although a higher *f* than that of the inline arrangement is observed at larger diagonal pitches. The highest TEF of 1.1 was recorded for the 45° oblique angle configuration. The triangular layout and the 30° oblique angle demonstrated favourable TEF values of 1.06 and 1.05, respectively.

## 5. Fouling resistance and particulate depositions in HX

The thermo-hydraulic efficiency of HX can be significantly enhanced by applying various innovative methods, as discussed above. These methods include altering the HT surfaces to be corrugated, using diverse types, patterns, and shapes of tubes, incorporating various fin types and patterns^27–41^ to reduce thermal resistance on the air side, employing nano-fluids^23^ as cooling mediums, vibrating the heat transfer surfaces with low-frequency vibrations, and utilizing swirl flow devices,^42^ among others. Despite these advancements, the performance of HX in terms of *η*_thm_ (thermal efficiency)and Δ*p* is significantly affected by issues such as fouling, corrosion, and erosion on the HT surfaces, which require considerable attention.

Fouling is characterized by the buildup of unwanted or harmful deposits on the heat transfer surfaces. Generally, it involves the accumulation and growth of materials that adversely affect thermal performance. The fouling process includes various heat, mass, and energy transfer phenomena associated with heat exchanger operations. Achieving improved TEF and enhanced HT characteristics while maintaining a low Δ*p* is crucial across many industrial sectors, including refrigeration, power generation, automotive, aerospace, process industries, cryogenics, waste gas heat recovery, seawater applications, technical fields, petrochemicals, and vegetable oil refining.

Developing a fouling layer on the HT surfaces acts as an insulating barrier, impeding HT from the liquid to the solid surface. This insulation effect reduces the flow area, leading to increased flow velocity for a given *v*_f_ rate, which in turn raises the Δ*p* and pumping power.

The complexity of fouling increases due to its dependence on various operating system variables. The fundamental fouling process can be categorized into initiation, transport, deposition, removal, ageing.^188^

Fouling has significant negative impacts on heat exchangers, including increased maintenance and operating costs, higher capital investment, production losses, energy inefficiencies, and remedial expenses. Fouling layers require frequent offline or online cleaning, which can be costly and time-consuming. Severe fouling often necessitates complete shutdowns for a thorough cleaning, impacting production and increasing machinery, labor, and standby equipment costs. Additionally, fouling reduces heat transfer efficiency, necessitating more fuel to achieve the desired performance and increasing costs due to higher pressure drops and potential damage to components.^189^

Researchers have focused on mitigating fouling through various techniques, including coatings, heat exchanger modifications, and advanced cleaning methods. A few of these techniques are listed below^190^

• The effect of material surfaces on reducing fouling resistance is well-established. Smooth surfaces tend to have a lower fouling rate than rough surfaces, while materials with high *kp* offer better fouling resistance. Areas on HT surfaces with low fluid velocity are more prone to particulate fouling. The design of fin-and-tube HX, including the transverse and longitudinal pitch of the tubes, significantly impacts particulate deposition. Increasing the longitudinal tube pitch leads to higher particle deposition due to the creation of low-velocity regions.

• Physical water treatment (PWT) techniques, such as electronic antifouling systems, the addition of fibers (from softwood or hardwood pulp), and catalytic materials (like zinc and tourmaline), play a key role in mitigating fouling and particulate buildup. Additionally, these additives can enhance HT performance by reducing fouling resistance.

• Louver fins generally have a higher fouling rate but also provide better HT performance than plane fins in fin-and-tube HX. In brazed plate HX, greater corrugation angles are associated with reduced fouling rates.

• Coatings on heat transfer surfaces can significantly repel fouling, improving heat transfer efficiency in heat exchangers.

## 6. Conclusions

A comprehensive evaluation is conducted to assess innovative techniques for enhancing HT performance and mitigating Δ*p* in HX based on researchers' experimental and numerical investigations. The influence of VGs on alleviating air-side thermal resistance and improving the thermodynamic characteristics of HX is extensively explored. The key concluding observations are as follows:

Protrusions from HT surfaces, such as VGs, play a prominent role in augmenting HT performance and enabling the design of HX with an even smaller area-to-volume ratio, lower thermal resistance on both air and liquid sides, and ultimately improved thermodynamic efficiency. Longitudinal VGs can induce three crucial HT mechanisms: secondary flow generation, boundary layer development, and intensification of fluid flow turbulence. The reduction in BL thickness is attributed to the primary and corner vortices generated by the longitudinal vortices of VGs. The poor HT region or wake region behind the tubes can be significantly diminished by the presence of VGs. They also can significantly enhance HT in the downstream region.

The precise location, dimensions, and attack angle of VGs are crucial to achieve substantial HT performance enhancement, albeit at the expense of moderate Δ*p*. However, the positioning of VGs has a negligible impact on Δ*p*. A staggered arrangement of winglets is responsible for higher HT rates than an inline configuration. Furthermore, DWPs are superior to RWPs, and winglets are preferable over wings in terms of higher HT rates and lower Δ*p*.

Longitudinal vortices induced by longitudinal VG, such as delta winglets, are superior to transverse vortices produced by transverse VG, at the cost of the same Δ*p*. Researchers have proposed various VG attack angles in their studies, with the optimal range being 30–45°.

Flow visualization investigations play a pivotal role in understanding fluid flow patterns at the microscopic level. The formation of horseshoe vortices, corner vortices, longitudinal vortices, and transverse vortices induced by VG can be easily analyzed and examined through these studies.

Fouling in heat exchangers can take various forms, including particulate, crystallization, biological, corrosion, chemical reaction, solidification, and combined fouling. These fouling layers cause flow obstructions and increase pressure drops, with gas-side fouling resistance being lower than liquid-side. Key factors affecting fouling include flow velocity, temperature, concentration, and surface materials. Higher flow rates reduce fouling resistance by generating shear stress on surfaces. Increased impurity concentrations lead to more significant particulate deposition, and temperature variations influence different types of fouling differently.

Smooth surfaces and materials with high thermal conductivity offer better fouling resistance. Low-velocity areas are more prone to fouling. In fin-and-tube heat exchangers, the longitudinal tube pitch affects particulate deposition, with higher pitches leading to increased deposition. Physical water treatment methods, such as electronic antifouling and using fibres or catalytic materials, help reduce fouling and enhance heat transfer. Louver fins generally have higher fouling rates but better heat transfer performance than plane fins. In brazed plate heat exchangers, higher corrugation angles reduce fouling. Surface coatings also effectively prevent fouling and improve heat transfer efficiency.

## Abbreviations

RWVG: Rectangular winglet vortex generator
RWPs: Rectangular wing pairs
DWP: Delta winglet pair
DWT: Delta-winglet tape
RTW: Rectangular trapezoidal winglet
RWP: Rectangular winglet pair
DWVG: Delta winglet VG
DW: Delta winglet
PR: Pitch ratio (*P*/*D*)
CARW: Curved angle rectangular winglet
TEF: Thermal enhancement factor
PCM: Phase change material
PFHS: Plate fin heat sinks
CFU: Common flow up
ARW: Angle rectangular winglet
CFU: Common flow up
MCW: Micro channel heat sink
CFD: Common flow down
SAH: Solar air heater
BL: Boundary layer
RW: Rectangular winglet
PFHS: Perforated fin heat sink
HT: Heat transfer
DSDW: double-sided delta-wings

### Symbols

*C*_d_
: Coefficient of drag
Δ*p*: Pressure drop
Ly: Transverse length
*h*_m_
: Convective heat transfer coefficient
Pe: Peclet number
Nu: Nusselt number
*S*_h_
: Sherwood number
*j*/*f*: Volume goodness factor
*R*_b_
: Blockage ratio
*R*_f_
: Fouling resistance
*f*
: Friction factor

### Greek symbols

β
: Attack angle/incidence angle
Λ
: Aspect ratio
Ω
: Goodness factor
ψ
: Horizontal rotation angle
e
: Ellipticity ratio
η
: Efficiency

## Data availability

No primary research results, software or code have been included and no new data were generated or analyzed as part of this review.

## Conflicts of interest

There are no conflicts to declare.
